# Transcription factor NR2F1 is involved in Parkinson’s disease

**DOI:** 10.4103/NRR.NRR-D-25-00290

**Published:** 2025-07-05

**Authors:** Annemarie de Vries, Silvia Bolognin

**Affiliations:** MERLN Institute for Technology-Inspired Regenerative Medicine, Maastricht University, Maastricht, the Netherlands

Nuclear receptor subfamily 2 group F member 1 (NR2F1, also called COUP-TF1) is a transcription factor and part of the steroid/thyroid hormone receptor superfamily (Gay et al., 2002). NR2F1 is an orphan receptor that dimerizes to bind DNA and acts as a repressor as well as an activator of the target genes (Gay et al., 2002; Bertacchi et al., 2019; Bonzano et al., 2023). It was found recently to regulate the transcription of mitochondrial genes and to affect the morphology and mass of mitochondria (Bonzano et al., 2023). NR2F1 is mainly known for its pleiotropic role in neurodevelopment, but it has recently been connected to neurodegeneration in the context of Parkinson’s disease (PD) (Walter et al., 2021). This perspective summarizes the known functions of NR2F1 and offers a new perspective on its potential role in neurodegeneration.

An important function of NR2F1 in the central nervous system is to guide neurodevelopment. Manipulations of NR2F1 expression levels during development led to altered neocortical patterning, cell migration, and cell fate determination (Alfano et al., 2013). NR2F1 was shown to be important for the differentiation of neurons versus astrocytes from neural stem cells (NSCs) (Bonzano et al., 2018). This key role of NR2F1 during brain development is reflected in its high expression during discrete temporal windows of brain development. *In vitro* studies demonstrated increased expression of *NR2F1*, specifically 15 days after the beginning of neuronal induction in NSCs (Gomez Ramoz et al., 2023). NR2F1 expression also persists in the adult brain. NR2F1 is expressed in the adult human cortex, interneurons, and pyramidal cells (Varga et al., 2015). However, its functions are unclear (Varga et al., 2015). The continued expression of NRF21 suggests a sustained functional role. NR2F1 has been shown in rodents to control the expression of tyrosine hydroxylase, which is involved in the production of dopamine, in adult neurons in the olfactory bulb (Bovetti et al., 2013).

Additional insights about the role of NR2F1 emerged from mutational studies. Induction of genetic loss-of-function of NR2F1 in radial glial cells of adult mice showed impaired neurogenesis, no changes in NSC/progenitor proliferation and newborn cell survival, and increased astrocyte development (Bonzano et al., 2018). Mutations in *NR2F1* led to upregulation of Hedgehog activators such as *GLI1* and downregulation of Hedgehog pathway repressors, together with enrichment in the promoter or enhancer region of those genes, which accounts for altered neuron differentiation of NSCs in the adult brain (Zhang et al., 2020). Mutations in the *NR2F1* gene are linked to a rare neurodevelopmental disorder called Bosch-Boonstra-Schaaf optic atrophy syndrome. Most of these mutations alter NR2F1 transcriptional regulatory activity. The most well-known variants are *de novo* and dominant mutations, without a familial component. These missense and nonsense variants result in premature protein truncation. Affected patients present multifaceted clinical symptoms that are often associated with visual impairment, intellectual disability, epilepsy, and autistic traits (Bonzano et al., 2023).

We observed that NR2F1 expression levels are altered in an *in vitro* model of PD (Walter et al., 2021), the second most common age-associated neurodegenerative disease after Alzheimer’s disease. While most PD cases are sporadic, several mutations are responsible for a familial form of PD. The most common mutation causing autosomal-dominant PD is G2019S in the leucine-rich repeat kinase 2 (LRRK2). Genome-wide association study analyses revealed that LRRK2 variants also contribute to the etiology of sporadic PD (Simón-Sánchez et al., 2009). Using induced pluripotent stem cells generated from PD patients carrying the LRRK2-G2019S mutation, we observed significant downregulation of *NR2F1* gene expression in neuroepithelial stem cells, dopaminergic neurons, and midbrain organoids compared with age-matched controls. Single-cell RNA sequencing analysis in midbrain organoids revealed that *NR2F1* is also downregulated in astrocytes, which are fundamental to preserving neuronal homeostasis. Altered NR2F1 expression can disrupt astrocyte differentiation, which in turn can lead to a chronic pro-inflammatory environment (Bonzano et al., 2018). Acute inflammation can further downregulate Nr2f1, as observed when lipopolysaccharide treatment in the adult mouse brain induced acute inflammation that downregulated Nr2f1 and upregulated glial markers (Bonzano et al., 2018). This observation stresses the importance of maintaining physiological NR2F1 expression levels to ensure proper astrocyte development and balance to provide a non-inflammatory environment. The introduction of the LRRK2-G2019S mutation into induced pluripotent stem cell models derived from healthy individuals also resulted in *NR2F1* downregulation. This result demonstrates that *NR2F1* downregulation is directly mediated by the LRRK2-G2019S mutation and not by the genetic background of the patients.

**Figure 1:**
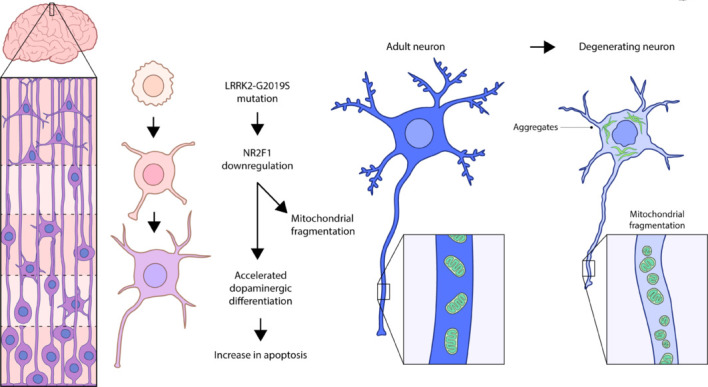
Potential roles of nuclear receptor subfamily 2 group F member 1 (NR2F1) from brain development to degeneration in Parkinson’s disease. NR2F1 is a key transcription factor that has been extensively studied for orchestrating the formation of the neocortex. However, its role and functions might be underestimated, as we observed NR2F1 downregulation in induced pluripotent stem cell–derived models carrying the Parkinson’s disease-associated mutation LRRK2-G2019S compared with controls (Walter et al., 2021). NR2F1 deletions were also associated with mitochondrial fragmentation in mouse models (Bonzano et al., 2023). As Parkinson’s disease is associated with cellular mitochondrial abnormalities, it is tempting to speculate that NR2F1 might contribute to dopaminergic degeneration by eliciting mitochondrial dysfunctions. These findings prompt future investigations about the potential involvement of NR2F1 in a neurodegenerative disease context.

We also showed the functional impact of the LRRK2-G2019S mutation on dopaminergic neurons (Walter et al., 2021). Compared to age-matched controls, dopaminergic neurons from PD patients carrying the LRRK2-G2019S mutation showed faster dopaminergic differentiation and faster cell cycle exit compared with controls. However, the differentiation of NSCs toward the dopaminergic lineage was characterized by increased cell death compared with controls (**Figure 1**). Accelerated dopaminergic neuron differentiation and increased cell death were also observed *in vivo* in Nr2f1-deficient mouse embryos compared with wild-type controls. This set of data suggests that decreased NR2F1 expression levels, a consequence of LRRK2-G2019S expression, result in an increased susceptibility of dopaminergic neurons to cell death. Considering the connection between NR2F1 and mitochondrial functions in the brain (Bonzano et al., 2023), it is tempting to speculate that the LRRK-G2019S and NR2F1 pathological interaction might involve abnormal expression of nuclear mitochondrial genes. Downregulation of *Nr2f1* in mice resulted in abnormal expression of nuclear-encoded mitochondrial genes and reduction in mitochondrial branching with increased fragmentation. Moreover, the expression of mitochondrial complexes I–V decreased in *Nr2f1*-knockout mice compared with controls. Only three of these complexes (I, II, and V) are direct targets of NR2F1 based on chromatin immunoprecipitation-sequencing data. This data also suggests the indirect action of NR2F1 on complexes III and IV (Bonzano et al., 2023).

The pathway through which LRRK2-G2019S expression affects NR2F1 is still unknown. Identifying and understanding the molecular players in this interaction would provide critical insights to map potential targets to halt the pathogenesis of PD. NR2F1 contains several putative phosphorylation sites, some of which have been proven to be phosphorylated *in vivo* by mitogen-activated protein kinase and protein kinase C (Gay et al., 2002). Our hypothesis posits a direct interaction between NR2F1 and LRRK2, with NR2F1 being a substrate of LRRK2. The G2019S mutation is located in the activation loop of the kinase subdomain and confers enhanced phosphorylation activity. Several transcription factors, including the nuclear hormone receptors, can be regulated by phosphorylation.

Further studies interrogating the interaction between NR2F1 and LRRK2 and offering detailed insights about perturbations resulting from gene expression can be performed with diverse approaches. While autoptic brain samples are the ideal specimens to test whether the interaction between NR2F1 and LRRK2 is relevant for aging and neurodegenerative conditions, patient-specific induced pluripotent stem cell-derived models can provide an innovative tool to perform disease modeling because they maintain the genetic background of the patients. Additionally, the interplay between glial cells and neurons can be modeled in a three-dimensional context using brain organoids. The continuous development of single-cell RNA sequencing and analysis pipelines facilitates mapping changes in gene expression in complex microtissues. Perturbations in gene regulatory networks can be identified with single-cell multi-omics, as was done in our case with the LRRK2-G2019S mutation. Furthermore, high-content automated image analysis enables the phenotypic assessment of organoids and microtissues to evaluate the impact of these perturbations.

The potential connection between the imbalance of transcription factors important for brain development and neurodegenerative disorders is not surprising. Similarities between neurodevelopmental and neurodegenerative disorders imply that defects during development render the brain more vulnerable to developing neurodegenerative diseases, such as PD, later in life. Defects in neurogenesis are also described in neurodegenerative diseases, highlighting the potential roles of transcription factors such as NR2F1 in age-associated conditions. A neurodevelopmental component has been posited for PD (Le Grand et al., 2015). The demonstrated role of NR2F1 in the adult olfactory bulb (Bovetti et al., 2013) is interesting because a loss of smell often precedes PD diagnosis. Furthermore, we recently showed that NR2F1 is part of the core regulatory circuit of neuroepithelial stem cells harboring the LRRK2-G2019S mutation, which showed disturbed dopaminergic neuron development and downregulation of *NR2F1* (Walter et al., 2021).

In summary, we believe the roles of the transcription factor NR2F1 in the adult and aging brain deserve further attention. Its established roles in astrogliogenesis, neurogenesis, and mitochondrial homeostasis, processes that are all altered in the pathology of PD, argue for its critical involvement in neurodegeneration. NR2F1 also connects mitochondrial dysfunction, described as an important contributor to PD pathology, to dopaminergic neurons. Because NR2F1 is important for neurodevelopment and is downregulated in developmental cell models carrying the PD-associated LRRK2-G2019S mutation, the developmental component of PD pathology is an important angle. Studying NR2F1 and the neurodevelopmental basis of PD can potentially give us new insights into its pathogenesis. Understanding what precedes the diagnosis of PD can lead to preventative measures and/or earlier interventions and a tremendous improvement in life quality. In the case of PD, 80% of dopaminergic neurons are lost before diagnosis, thus, earlier intervention is a key aspect of symptom management, considering that PD currently has no disease-modifying therapy.
